# Supplementary material for: Evaluating the relationship between negative foot speed and sprint performance using shoe-mounted inertial sensors
Source: PLoS One. 2024 Dec 20;19(12):e0303920. doi: 10.1371/journal.pone.0303920 (PMC11661630; doi:10.1371/journal.pone.0303920)
Supplement: Attachment — Submitted filename: Response to reviewers_vFinal.docx [file pone.0303920.s001.docx]

**Reviewer 1: GENERAL COMMENTS

This original study is interesting and addresses a key point for sports performance analysis: field measurements of sprint running mechanics using wearable sensors. The study has a limited scope, focusing on a single but important variable (negative foot speed at ground contact) and some limitations well acknowledged by the authors, but it brings a significant amount of actionable information for practitioners. I have specific comments I’d like the authors to address before acceptance can be recommended.**

Thank you so much for your feedback. Please see our responses to your comments below. Please note that we have added line numbers to the manuscript and refer to those, while the original feedback was based on the line numbers generated in the submission system.

Please note that in the previous version of the study, we focused solely on the vertical (VT) and anteroposterior (AP) components of negative foot speed. In the current version, we have included the magnitude of the negative foot speed vector. As a result, we have modified lines 29, 32, 105, 107, 108, 114, 196, 198, 203, 216 (Table 2), 223, 224, 227 (Table 3), 237, 254, 255, 258 (Table 4), 268, 275, 277, 280, 282, 285, 332, 348, 362 to reflect these changes. **1. Title: maybe “sprint technique” should be replaced by something more accurate and specific of what has been actually measured/studied. Running speed and negative foot speed or kinematics?**

Thank you for pointing this out. In lines 3-4 we have modified the title from “Assessing sprint technique with shoe-mounted inertial sensors” to “Evaluating the relationship between negative foot speed and sprint performance using shoe-mounted inertial sensors”.

**2. The paper is overall very well written, and the introduction is well structured. I think that the “ZUPT” approach should be briefly summarized in the intro or in the methods, so that the readers understand the concept without having to read an external reference.**

Thank you for your suggestion. In lines 59-66 we have added: “The ZUPT method [20, 22] includes four primary steps. The stride segmentation step employs raw IMU signals to identify time points of zero velocity, indicating when the velocity of the foot is approximately zero and is relatively stationary on the ground. The rotational orientation estimation step uses an Extended Kalman filter to resolve local, sensor-fixed, IMU measurements into the global inertial frame. The translational velocity estimation step integrates linear accelerations between two consecutive zero-velocity time points and corrects for integration drift error exploiting the assumption that the foot’s velocity is zero at each zero-velocity time point. The trajectory formation step determines stride parameters (e.g., stride length) by integrating foot velocities to obtain foot position.

**3. Line 78: causal/prediction terms should be used with caution in the context of a correlational design, it is preferable to say “strongly associated with sprint performance”.**

Thank you for pointing this out. In line 94 we have changed “is a strong predictor of sprinting performance” to “is strongly associated with sprinting performance”.

**4. Line 84: same comment, maybe use “would be associated with “rather than “be achieved with”.**

Thank you for pointing this out. In line 116 we have changed “would be achieved with” to “would be associated with”.

**5. In the results, it could be useful to comment on the magnitude of the correlations/relationships and not only on their significant/non-significant feature. If two correlations are significant, a moderate one and a large one should be interpreted differently.**

Thank you for your suggestion. In lines 204-206 we have added “Correlations below 0.50 were considered weak, correlations between 0.50 and 0.80 were considered moderate and correlations above 0.80 were considered strong”. In lines 218, 220 and 223 we have added “weak”.

**6. Line 181: for example, these are significant but rather low correlations, so just mentioning that these variables were “significantly correlated” is misleading.**

Thank you for your suggestion. In line 275 we have changed “significantly” to “weakly”.

**7. Line 188: I would take this relationship with caution, since the design of this study and others is mainly correlational. So, a “chicken and egg” scenario is possible: are faster athletes running with a more negative foot speed (and the correlation between negative foot speed and running speed is coincidental…) or are they running fast because of a negative foot speed? What counterarguments do we have to ascertain that it’s not a “correlation vs causation” issue, and that a more negative foot speed actually causes (at least partly) a faster running speed?**

Thank you for your comment. We agree that we cannot determine whether the relationship between negative foot speed and top sprinting speed is causal or coincidental. However, based on our conversations with coaches, they seem to believe that the relationship is causal.

In lines 309-320 we have added “A novel coaching approach involves athletes using foot-mounted IMUs in training to receive instant feedback on their negative foot speed. This allows them to actively focus on improving this aspect of their performance, potentially leading to faster sprinting speeds. Despite these advantages, it is essential to note that while our findings indicate a significant relationship between negative foot speed and peak running speed, our study design is correlational. Thus, we cannot definitively establish causality between these variables. A “chicken and egg” scenario is possible: are faster athletes running with a more negative foot speed, or is this relationship coincidental? Coaches, however, generally believe the relationship is causal [28,30]. They emphasize negative foot speed as a key sprinting technique to improve speed, suggesting that a more negative foot speed may reduce braking forces and allow for greater acceleration. Although further experimental research is needed to confirm a direct causal link, these insights provide a plausible explanation for why improving negative foot speed could lead to faster sprinting speeds.”

**8. Line 195: it is very interesting that shoe-mounted IMUs open such possibilities, but the authors should better clarify the amount of extra data analysis work typical users will have to perform to obtain the same measurements. Is the typical user without raw data processing skills able to reproduce what is shown in this study? Some comments should be made on this point within the discussion.**

Thank you for your suggestion. In lines 341-346 we have added “Third, the process of extracting metrics from raw IMU data involves complex data analysis steps, such as the rotational orientation estimation step. Users without advanced data processing skills may find these tasks challenging. Although some software platforms offer pre-processed metrics or simplified workflows, additional data analysis might still be necessary, highlighting the need for more accessible software tools to ensure usability for coaches and practitioners.

**9. Ket point: Figure 3 shows a significant yet pretty low correlation. The range of running speed is very wide: from 7.2 to 9.2 m/s. There is a gap without data between a group of “slow participants” maxing around 7.7 m/s and a group of faster participants running faster than 8.4 m/s. If one considers only the faster athletes (points above 8.4 m/s) this correlation seems to drop to very low, likely not significant. So, the conclusions of the study will be likely reversed when focusing only on faster athletes. This clearly changes the overall message. Could you please comment on that, and add this point to the limitations of the study (based on the correlation on the faster points only)? This is very clear in Figure 3 so any careful reader will notice that the general conclusion drawn from the full set of data does not apply to fast participants, which is a clear limitation of the approach.**

Thank you for pointing this out. The literature has established a clear relationship between horizontal foot velocity and speed [11,26,27], which the overall results of our study using IMU data confirm. As there are many factors that impact sprint speed, it is not surprising that increasing negative foot speed is not critical to run faster. However, our study does support the overall conclusion that negative foot speed is correlated to top sprint speed for a sample of sprinters. A larger sample of sprinters would be necessary to make a conclusion about top sprinters. In lines 335-341 of the discussion section, we have added “Figure 3 shows a significant yet relatively low correlation, particularly for the participants that ran faster than ~8.4 m/s. These differences could be due to small but expected errors in the calculation of foot speed [20, 24] or in our estimate of the body COM velocity. Studies of Olympic sprinters [32] have established that foot velocity is correlated to sprinting velocity. Therefore, measurements of additional participants who can sprint at speeds greater than 8.4 m/s are needed to determine if the results for our fastest participants are due to limitations in our measurement approach or due to individual differences in sprint technique”.

**Reviewer #2: This study deals with the investigation of correlations between negative foot speed and sprint performance. I suggest the acceptance of this work only if all the following points will be addressed.**

Thank you so much for your feedback. Please see our responses to your comments below. Please note that we have added line numbers to the manuscript and refer to those, while the original feedback was based on the line numbers generated in the submission system.

Please note that in the previous version of the study, we focused solely on the vertical (VT) and anteroposterior (AP) components of negative foot speed. In the current version, we have included the magnitude of the negative foot speed vector. As a result, we have modified lines 29, 32, 105, 107, 108, 114, 196, 198, 203, 216 (Table 2), 223, 224, 227 (Table 3), 237, 254, 255, 258 (Table 4), 268, 275, 277, 280, 282, 285, 332, 348, 362 to reflect these changes.

**Introduction**

**1. Please expand the description of the state-of-the-art of the instrumentations used to evaluate sprint performance both indoor and on track (e.g., pressure insoles, photo-cell systems, high-speed cameras, GPS, emg, etc.).**

Thank you for your suggestion. In lines 43-46 we have included more devices used to evaluate sprint performance: “Traditional approaches to quantify sprint performance technique include force plates [4,5,6,7], motion capture systems [9,11], optical measurement systems [12], high speed cameras [13], smartphone applications [13], resistance devices [14,15], GPS [16] and pressure insoles [17].

We have modified the references and the references section accordingly.

**2. Which sprint determinants can be estimated with MIMUS? With which accuracy? For which clinical (post-injury) and sportive (performance assessment) applications?**

In our previous study for sportive applications [20] we found that for speeds up to 9.5 m/s, cumulative and individual stride distance errors (comparing IMU estimates and camera-based estimates) were within -4 to 2% and -6 to 3%, respectively. Foot-worn IMUs can also provide highly accurate measures of instantaneous foot linear acceleration, angular velocity, foot orientation, and linear velocity. Position estimates from IMUs are the least accurate, as the calculation introduces the most integration drift error. The ZUPT approach has been applied across a wide range of clinical [36] and sportive [37].

[20] G. Aristizábal Pla, D. N. Martini, M. V. Potter, and W. Hoogkamer, “Assessing the validity of the zero-velocity update method for sprinting speeds,” *PLoS One*, vol. 19, no. 2, p. e0288896, Feb. 2024, doi: 10.1371/journal.pone.0288896.

[36] Rebula JR, Ojeda LV, Adamczyk PG, Kuo AD. Measurement of foot placement and its variability with inertial sensors. Gait Posture. 2013 Sep;38(4):974-80.

[37] Cain SM, McGinnis RS, Davidson SP, Vitali RV, Perkins NC, McLean SG. Quantifying performance and effects of load carriage during a challenging balancing task using an array of wireless inertial sensors. Gait Posture. 2016 Jan;43:65-9.

**3. Please explain better the relevance and the inertial-based methods to estimate the center of mass speed and its linkage with mentioned ZUPT method, force and power.**

Thank you for your suggestion. We have modified lines 68-74 to “The ZUPT method allows for the calculation of stride velocities, stride lengths, and stride times. Stride lengths can be divided by stride times to obtain estimates of the center of mass (COM) speed, as the average COM speed during a stride must be equivalent to (or very close to) the stride speed. Calculation of stride speeds during a sprint yields a velocity curve [20, 23, 24, 25]. From the velocity curve, sprinting performance determinants such as top speed [4,5,6,7] can be extracted. Additionally, the method proposed by Samozino et al. [22] can use split times from the velocity curve as inputs to obtain kinetic determinants of sprint performance, such as the ratio of force. “

**4. Provide a summary of the most widespread approached chosen by coaches to enhance sprint and running performance. Which is the biomechanical explanation of choosing to focus on increasing the negative foot speed?**

Before conducting this study, we interviewed over 30 coaches from different sports (e.g., football soccer, track) and the most widespread approach to enhance sprint and running performance was based on measures from the speed curve. For example, football coaches wanted to get acceleration/deceleration metrics while sprint coaches focused more on top speed to improve sprint performance.

There is not a biomechanical explanation of choosing to focus on increasing negative foot speed. Based on our conversations with coaches, they seem to believe that the relationship between negative foot speed and top speed is causal, and that is why we choose to focus on this variable.

In lines 309-320 we have added “A novel coaching approach involves athletes using foot-mounted IMUs in training to receive instant feedback on their negative foot speed. This allows them to actively focus on improving this aspect of their performance, potentially leading to faster sprinting speeds. Despite these advantages, it is essential to note that while our findings indicate a significant relationship between negative foot speed and peak running speed, our study design is correlational. Thus, we cannot definitively establish causality between these variables. A “chicken and egg” scenario is possible: are faster athletes running with a more negative foot speed, or is this relationship coincidental? Coaches, however, generally believe the relationship is causal [28,30]. They emphasize negative foot speed as a key sprinting technique to improve speed, suggesting that a more negative foot speed may reduce braking forces and allow for greater acceleration. Although further experimental research is needed to confirm a direct causal link, these insights provide a plausible explanation for why improving negative foot speed could lead to faster sprinting speeds.”

**5. Which is the novelty of this work? Which lack in the literature is filled? Which are the differences with respect to previous studies investigating the relationship between the anteroposterior foot speed and the peak running speed?**

The novelty of the work is the implementation of IMUs in an ecologically relevant environment (on an outdoor track) to quantify the relationship between negative foot speed and top sprinting speed. Our study addresses a gap in the literature by demonstrating that IMUs can provide actionable metrics, such as negative foot speed, in addition to assessing sprinting performance, as shown in our previous study.

While prior studies relied on motion capture systems and high-speed cameras to investigate the relationship between negative foot speed and top sprinting speed, we demonstrate that a similar analysis can be conducted with IMUs. Additionally, limited research has focused on the vertical (VT) component and the magnitude of the negative foot speed vector as predictors of sprinting performance.

We stressed the limitations we are overcoming with our study in the following:

- Lines 93-96 with “Therefore, the AP component of negative foot speed, measured with different devices and in different running environments, is strongly associated with sprinting performance [11,29,30]. Unfortunately, no studies have examined the relationship between negative foot speed and top sprinting speed with shoe-mounted IMUs “.

- Lines 99-101 with “Therefore, more studies examining the VT component of negative foot speed are needed to better assess the VT component of negative foot speed as being a predictor of sprinting performance.”

- Lines 102-109 with “While previous studies [11,29,30] have primarily focused on the AP and VT components of negative foot speed, it is important to recognize that these components represent only part of the overall motion. By examining the complete velocity vector at touchdown, which includes both AP and VT components (i.e., the vector magnitude), we can potentially gain a more comprehensive understanding of sprinting dynamics. Unfortunately, no studies have ever tried to examine the negative foot speed vector magnitude as being a predictor of sprinting performance. Therefore, more studies examining the magnitude of the negative foot speed are needed to better assess its potential as a predictor of sprinting performance.”.

**6. Provide a literature context also for vertical foot speed correlations with sprint determinants. Why did you choose to study this variable if only one study found significant correlations with peak sprint speed?**

Thank you for your suggestion. We have provided a literature context for the vertical component of negative foot speed in lines 97-101 with “Only one study [26] examined the relationship between the vertical (VT) component of negative foot speed (i.e., the global velocity vector at touchdown) and peak sprinting speed but did not find significant correlations. Therefore, more studies examining the VT component of negative foot speed are needed to better assess the VT component of negative foot speed as being a predictor of sprinting performance.”.

We chose to study this variable because coaches often emphasize that at the end of every stride in a sprint, the foot must move downward and backward more quickly. However, only one study has specifically investigated the vertical component of negative foot speed in relation to top sprinting speed.

**7. End the introduction with the clear explanation of the aims of the study.**

Thank you for your suggestion. We have modified lines 93-118 to end with a clear explanation of the aims of the study in lines 110-118 with “The main aim of this study was to use IMUs to investigate the relationship between negative foot speed and peak sprinting speed attained during an 80-meter sprint on an outdoor track. Specifically, we wanted to investigate if (1) the AP component of negative foot speed could be associated with peak sprinting speed; (2) the VT component of negative foot speed could be associated with peak sprinting speed and; (3) the magnitude of the negative foot speed vector could be associated with peak sprinting speed. Based on optical motion capture or high-speed camera studies [11,25,26], we hypothesized that faster peak speeds would be associated with greater AP components of negative foot speed. The secondary aim of this study was to investigate the relationship between negative foot speed and stride speed across all strides during the 80-meter sprint.”

**Methods**

**8. Does the implemented method for IMU axes re-orientation guarantee that the anteroposterior axis is always aligned with the primary anatomical foot axis?**

No, it does not guarantee perfect alignment, as the direction of the ML and AP axes are dependent on the orientation of the IMU relative to the anatomical axes of the foot. However, this re-orientation does not impact the results presented in the manuscript because our analyses are done using velocity and position data that is represented in the global inertial reference frame that is immune to slight differences in our definitions of the AP and ML axes relative to the IMU.

In line 149 we have added “assumed anatomical directions”.

**9. How the coordinate frame is rotated to be constantly aligned with the direction of progression? How was the yaw angle defined to correct the anteroposterior axis?**

To ensure the coordinate frame was consistently aligned with the direction of progression, we first obtained the 3D position of the foot in a global inertial reference frame where the Z axis was aligned with gravity. To account for potential yaw drift, we then defined a stride reference frame for each stride that points from foot contact at the start of the stride to foot contact at the end. The direction is then defined as follows:

$$\theta= \tan^{-1} \frac{p_{x, t=end}}{p_{y,t=end}}$$

(4)

where $p_{x, t=end}$ and $p_{y,t=end}$ are the displacements of the foot over time, and t=end represents the x or y displacement at the last time point (the end of the stride). This angle was then used to define a rotation matrix:

$$R_{VT}=\left[ \begin{matrix} cos(\theta) & -sin(\theta) & 0 \\ sin(\theta) & cos(\theta) & 0 \\ 0 & 0 & 1 \end{matrix} \right]$$

(5)

which is applied to resolve the foot displacement and velocity in a stride-aligned reference frame (i.e., the global inertial reference frame). Therefore, following this rotation, for a given stride, the AP-axis of the stride reference frame was defined by the displacement of the foot during the stride (i.e., by the direction of running) and the VT-axis was aligned with gravity.

We have added this explanation in 174-190.

**10. Why did you define the initial contact instants with the mentioned definition based on vertical acceleration? Mention the reference paper(s).**

We defined the initial contact instants as time points when the VT acceleration changed from negative to positive. This definition, included in lines 165-166, represents the moment when the foot is slowing down because it’s contacting the ground. Our definition is based on the physics of sprinting (the foot will slow down after making contact with the ground) and is similar to definitions used by others [35].

[35] C. Maiwald, T. Sterzing, T.A. Mayer & T.L. Milani (2009) Detecting foot-to-ground contact from kinematic data in running, Footwear Science, 1:2, 111-118.

**11. Were the performance of identification of initial contact always accurate despite great change in running speed to due initial sprint accelerations?**

We are confident in the consistent and correct identification of initial contact given our definition of initial contact (detailed in the previous response). We cannot speak to the accuracy of initial contact because we have no ‘ground-truth’ ground contact data to compare to. Our detection of initial contact was not done by a fully-automated algorithm. Due to the rapid changes in running speed and initial sprint accelerations, we found it challenging to detect initial contacts reliably. To address this, we manually selected the maximal peaks in the acceleration magnitude and then automatically identified zero crossings in the vertical (VT) acceleration signal.

We have added this explanation in lines 167-170.

**12. Consider to better explain the method to estimate the velocity stride-by-stride.**

Thank you for pointing this out. Please see our response to your comment 3.

**13. Readers may be interested in looking at correlations between the analyzed inertial-based parameters with stride-by-stride foot velocity, not only the peak speed within the sprint. Can you provide these analyses?**

Thank you for your suggestion. We have modified lines 26-32 of the abstract with the following: “The secondary aim of this study was to use IMUs to investigate the relationship between contact foot velocity and running speed on a stride-by-stride basis for a sprint on an outdoor track. … … For the mean peak stride speed of 7.98±0.78m/s and average stride speed of 7.43±0.68m/s, the adjusted R^2^ values were 0.27 and 0.69, 0.42 and 0.64, and 0.42 and 0.75 versus the anteroposterior, vertical, and magnitude of initial contact foot velocity, respectively.”.

We have modified lines 116-118 of the introduction with the following: “The secondary aim of this study was to investigate the relationship between the velocity of the foot at initial contact and stride speed across all strides during the 80-meter sprint.”

We have modified lines 195-198 of the methods section with the following: “(i.e., top stride speed, global VT velocity vector at initial contact for the top speed stride, relative AP velocity vector for the top speed stride, velocity vector magnitude at the top speed stride, stride speeds for all strides, global VT velocity vector for all strides, relative AP velocity vector for all strides and velocity vector magnitude for all strides).” In lines 202-203 we also replaced “top stride speeds” with “stride speeds”.

We have added a section in the results “Relationships between initial contact foot velocity and sprinting speed“, and in lines 218-225 we added the following: “The relative AP velocity vector had a moderately significant relationship to peak stride speed. Faster speeds were achieved with greater AP components of the relative foot velocity vector at initial contact (R^2^=0.69; p<0.001; y=-0.99-0.52x [where x denotes stride speed]; Fig 6; Table 4). The global VT velocity vector had a moderately significant relationship to stride speed. Faster speeds were achieved with greater VT components of the global velocity vector (R^2^=0.64; p<0.001; y=-0.83-0.25x [where x denotes stride speed]; Fig 7; Table 4). The velocity vector magnitude had a moderate significant relationship to stride speed. Faster peak speeds were achieved with greater velocity vector magnitudes (R^2^=0.75; p<0.001; y=1.44+0.56x [where x denotes stride speed]; Fig 8; Table 4).”

We have added the tables and the figures to the results section.

We have modified the discussion section in lines 277-282 with the following: “The secondary aim of this study was to investigate the relationship between the velocity of the foot at initial contact and stride speed across all strides during the 80-meter sprint. We found that the AP, VT and magnitude components of the velocity of the foot at initial contact were moderately correlated to stride speed. Specifically, for stride speeds of 7.43±0.68m/s the adjusted R^2^ values were 0.69, 0.64 and 0.75 for the AP, VT and magnitude components of the velocity of the foot at initial contact, respectively.”

**Results**

**14. At which percentage of 80-m sprint the speed peak occurred? Was it consistent among runners?**

Peak speed occurred between 20 and 70m for the 80m sprint for all runners. Please find below a figure that includes the velocity curves for all runners with the peak speeds circled in red. Further, the literature confirms that the maximal speed is recorded between 20-70m (Slawinski J, Termoz N, Rabita G, Guilhem G, Dorel S, Morin JB, Samozino P. How 100-m event analyses improve our understanding of world-class men’s and women’s sprint performance. Scand J Med Sci Sports. 2017;27(1):45–54).

We have reported that peak speed occurred between 20 and 70m for the 80m sprint for all runners in lines 213-214 of the results section.

**15. Were the variable distributions normal?**

Yes, the variable distributions were normal. We considered the variables to be normally distributed if the skewness values were between -1 and +1. For the vertical component of negative foot speed, the skewness was -0.18. For the AP component of negative foot speed, the skewness was -0.86. For the peak speed, the skewness was -0.48. For the vector magnitude, the skewness was 0.45.

We added this to lines 200-201: “We first confirmed that all the variables were normally distributed (i.e., −1 < accepted skewness < 1).”

**16. How were relative anteroposterior velocity calculated? Which is the differences with the global ones?**

The relative anteroposterior (AP) velocity was calculated by subtracting the center of mass (COM) speed from the global AP velocity. In contrast, the global vertical velocity vector does not require any subtraction, as it directly represents the vertical component without needing to account for the COM speed.

**17. Was the method used to assess foot velocity validated? Provide information in terms of accuracy in the estimation of imu-based foot speed.**

Yes, in our previous study [20], we validated the ZUPT method in estimating total distances traveled and stride lengths. For speeds up to 9.5 m/s, cumulative and individual stride distance errors were within -4 to 2% and -6 to 3%, respectively. While we do not have (nor are we aware of) comparisons of instantaneous foot velocity to another system (e.g. MOCAP), we are confident that velocities calculated using our approach will be more accurate than our position calculations. This is because velocity calculations are corrected using the ZUPT approach and do not contain the additional integration drift error that position calculations contain.

[20]	G. Aristizábal Pla, D. N. Martini, M. V. Potter, and W. Hoogkamer, “Assessing the validity of the zero-velocity update method for sprinting speeds,” *PLoS One*, vol. 19, no. 2, p. e0288896, Feb. 2024, doi: 10.1371/journal.pone.0288896.

**18. The results subtitle ‘Effect of sprinting speeds and body mass on stride length estimation’ is not pertinent. Add the analysis of the effect of the sprint speed (averaged within 80 m) and the body mass on negative AP and V speeds and also on stride length, which are missing.**

Sorry for the confusion. We did not intend to assess the effects of sprinting speeds and body mass on stride length estimation in this study. We did this for our previous study. Apologies for our mistake. We changed the title in lines 210-211 from “Effect of sprinting speeds and body mass on stride length estimation” to “Relationships between initial contact foot velocity and top sprinting speed”.

**19. Results should be improving providing a more detailed picture of the numerical findings of the work, which are now very limited. A table or diagram showing the obtained peak negative speeds along the three anatomical axis, the peak speed, the averaged speed, and the stride length would be really helpful.**

Thank you for your suggestion. We have added the following table (table 2) to the results section in line 216:

| ID number | Stride length (m) | IMU estimated top speed (m/s) | IMU estimated average speed (m/s) | VT negative foot velocity (m/s) | AP negative foot velocity (m/s) | Magnitude foot velocity (m/s) |
| --- | --- | --- | --- | --- | --- | --- |
| 1 | 4.08 | 7.44 | 6.61 | -2.23 | -4.92 | 5.41 |
| 2 | 4.18 | 8.35 | 7.23 | -2.90 | -5.64 | 6.34 |
| 3 | 4.27 | 8.60 | 7.60 | -2.79 | -5.73 | 6.38 |
| 4 | 3.40 | 7.18 | 6.35 | -2.91 | -4.71 | 5.54 |
| 5 | 3.53 | 7.89 | 6.79 | -2.68 | -4.94 | 5.62 |
| 6 | 4.17 | 8.93 | 8.00 | -3.62 | -6.45 | 7.39 |
| 7 | 4.38 | 9.20 | 8.03 | -3.01 | -5.14 | 5.96 |
| 8 | 4.03 | 7.88 | 7.15 | -2.85 | -5.15 | 5.89 |
| 9 | 4.07 | 8.49 | 7.67 | -2.97 | -5.14 | 5.94 |
| 10 | 3.76 | 7.23 | 6.48 | -2.39 | -4.19 | 4.82 |
| 11 | 3.99 | 8.44 | 7.57 | -2.64 | -5.17 | 5.81 |
| 12 | 3.91 | 8.60 | 7.69 | -3.10 | -5.71 | 6.49 |
| 13 | 3.77 | 8.80 | 7.93 | -2.53 | -6.96 | 7.40 |
| 14 | 3.95 | 9.37 | 8.45 | -3.35 | -5.06 | 6.07 |
| 15 | 3.90 | 8.56 | 7.62 | -2.71 | -4.99 | 5.68 |
| 16 | 3.95 | 8.74 | 7.78 | -3.20 | -5.24 | 6.14 |
| 17 | 4.35 | 9.34 | 8.20 | -3.47 | -5.73 | 6.70 |

The old table 2 is now named table 3 in lines 220, 223, 225 and 227.

**20. Adding a figure depicting the foot speed with respect to time (or 0-80 m) along three anatomical axes is recommended.**

Thank you for your suggestion. We have added the figure below in lines 241-247:

The figure is introduced in lines 241-242 with the following “Figure 6 depicts the velocity vector at touchdown with respect to distance along the three anatomical axes for a representative subject (P01).”

We included the figure description in lines 244-247 with the following: “Fig. 6. IMU-derived measures for an 80-meter sprint on a stride-by-stride basis as a function of the distance traveled for a representative subject (P01). (A) Speed versus cumulative stride length. (B) Decomposition of foot velocity into anteroposterior (blue), vertical (black), and mediolateral (red) components.”

**Discussion**

**21. This section can be improved. Stress the relevance of monitoring the negative AP and V peak speed in sprinting to improve running performance. Why the correlated peak speed are important?**

Thank you for your suggestion. There is not a biomechanical explanation of choosing to focus on increasing negative foot speed. Based on our conversations with coaches, they seem to believe that the relationship between negative foot speed and top speed is causal, and that is why we choose to focus on this variable.

In lines 309-320 we have added “A novel coaching approach involves athletes using foot-mounted IMUs in training to receive instant feedback on their negative foot speed. This allows them to actively focus on improving this aspect of their performance, potentially leading to faster sprinting speeds. Despite these advantages, it is essential to note that while our findings indicate a significant relationship between negative foot speed and peak running speed, our study design is correlational. Thus, we cannot definitively establish causality between these variables. A “chicken and egg” scenario is possible: are faster athletes running with a more negative foot speed, or is this relationship coincidental? Coaches, however, generally believe the relationship is causal [28,30]. They emphasize negative foot speed as a key sprinting technique to improve speed, suggesting that a more negative foot speed may reduce braking forces and allow for greater acceleration. Although further experimental research is needed to confirm a direct causal link, these insights provide a plausible explanation for why improving negative foot speed could lead to faster sprinting speeds.”

**22. Expand the comparison with the literature in terms of assessing sprint performance with wearable technology.**

Thank you for your suggestion. We have expanded a comparison with the wearable literature for assessing sprint performance in lines 301-306 with: “Furthermore, when compared to other wearable technologies like GPS devices [16] or pressure insoles [17], shoe-mounted IMUs offer superior accuracy in capturing detailed foot mechanics crucial for sprint performance. GPS units, while useful for tracking overall sprint velocity and distance, lack the accuracy needed to assess sprinting speed on a stride-by-stride basis. Pressure insoles, although informative for plantar pressure distribution, do not capture key metrics like foot speed and acceleration as comprehensively as IMUs.”

**23. Lines 203-204: provide a more comprehensive comparison with previous studies assessing stride length, stride velocity and/or contact time through IMUs.**

Thank you for your suggestion. We have incorporated a more comprehensive comparison with previous studies in lines 323-330 by “Previous work has validated other kinematic quantities obtained from shoe-mounted IMUs during sprinting [20, 21, 28, 29]. Aristizábal Pla et al. 2024 [20] and de Ruiter et al. 2022 [21] validated IMU-based stride lengths with a camera-based system. Though, Aristizábal Pla et al. 2024 [20] obtained an average bias ± limits of agreement of -0.27±4.61% while de Ruiter et al. 2022 [21] obtained an average bias ± limits of agreement of -2.51±8.54%. de Ruiter et al. 2022 [28] and van den Tillaar et al. 2021 [29] validated IMU-based step detection algorithms with optical measurement systems and force plates, respectively. de Ruiter et al. 2022 [28] obtained a bias of 0.43 ms with limits of agreement of -6.40 ms while van den Tillaar et al. 2021 [29] obtained a correlation coefficient of 0.044.”.

**24. Relevance of the work, possible scenarios of the applications of the main findings and study limitations should be stressed to provide a more complete Conclusion section.**

Thank you for your suggestion. We have expanded the conclusion section in lines 347-357 by: “In conclusion, the results of this study support the use of IMUs for quantifying sprinting technique with actionable metrics, including the AP component, VT component, and magnitude of negative foot speed. Our results demonstrate that higher values of these components are associated with faster peak sprinting speeds, corroborating coaching practices that emphasize the "pawing" or "shipping" foot action. While IMUs offer significant advantages such as affordability, ease of setup, minimal interference with movement, and real-time feedback, several limitations must be addressed. These include the absence of comparison with optical motion capture systems and the potential challenges in data processing for users lacking advanced technical skills. Future research should focus on validating these findings against gold-standard methods and improving the accessibility of IMU-based analysis. Despite these challenges, the insights gained from this study suggest that IMUs hold substantial potential for enhancing sprint performance analysis and coaching methodologies.”

**25. Which are the novelties in suggestions in coaching approaches or in sprint performance enhancement techniques?**

The novelties in suggestions in coaching approaches are that athletes use foot-mounted IMUs in training to get instantaneous feedback on their negative foot speed, allowing them to actively work on improving their negative foot speed, which could result in faster sprinting speeds.

We have added this in lines 309-311.

**Title**

**26. Consider to rephrase the title since this study did not generally assess sprint techniques, but much more specifically analyzed the correlations between foot negative and peak speeds.**

Thank you for pointing this out. In lines 3-4 we have modified the title from “Assessing sprint technique with shoe-mounted inertial sensors” to “Evaluating the relationship between negative foot speed and sprint performance using shoe-mounted inertial sensors”.
